# Perioperative mortality among geriatric patients in Ethiopia: a prospective cohort study

**DOI:** 10.3389/fmed.2023.1220024

**Published:** 2023-11-02

**Authors:** Amanuel Sisay Endeshaw, Misganew Terefe Molla, Fantahun Tarekegn Kumie

**Affiliations:** Department of Anesthesia, College of Medicine and Health Science, Bahir Dar University, Bahir Dar, Ethiopia

**Keywords:** geriatrics, surgery, outcome, postoperative mortality, Ethiopia

## Abstract

**Background:**

With the dramatic growth in the aged population observed in developed and developing nations, the older population burdened by unmet demand for surgical treatment has become a significant yet unnoticed public health concern in resource-limited countries. Studies are limited regarding surgical mortality of geriatric patients in Africa. Therefore, this study aims to estimate the incidence and identify predictors of postoperative mortality using prospective data in a low-income country, Ethiopia.

**Methods and materials:**

A prospective cohort study was conducted from June 01, 2019, to June 30, 2021, at a tertiary-level hospital in Ethiopia. Perioperative data were collected using an electronic data collection tool. Cox regression analysis was used to identify predictor variables. The association between predictors and postoperative mortality among geriatrics was computed using a hazard ratio (HR) with a 95% confidence interval (CI); *p*-value <0.05 was a cutoff value to declare statistical significance.

**Results:**

Of eligible 618 patients, 601 were included in the final analysis. The overall incidence of postoperative mortality among geriatrics was 5.16%, with a rate of 1.91 (95% CI: 1.34, 2.72) deaths per 1,000 person-day observation. Age ≥ 80 years (Adjusted hazard ratio (AHR) = 2.59, 95% CI: 1.05, 6.36), ASA physical status III/IV (AHR = 2.40, 95%CI 1.06, 5.43), comorbidity (AHR = 2.53, 95% CI: 1.19, 7.01), and emergency surgery (AHR = 2.92, 95% CI: 1.17, 7.27) were the significant predictors of postoperative mortality among older patients.

**Conclusion:**

Postoperative mortality among geriatrics was high. Identified predictors were age ≥ 80 years, ASA status III/IV, comorbidity, and emergency surgery. Target-specific interventions should be addressed to improve high surgical mortality in these patients.

## Introduction

According to World Health Organization’s global forecast, 1 in 6 people will be 60 years and over by 2030; in 2022, the world population aged 60 years and over is estimated to be 1.4 billion, which was 1 billion in 2020 and predicted to be double (2.1 billion) by 2050 (1). A drastic rise in the elderly population is observed in developed and developing countries, constituting 20% of the general population in developed and 10% in developing countries; the projection for the coming consecutive decades is expected to increase rapidly (2). In the context of underdeveloped countries, between 2000 and 2030, the elderly population in sub-Saharan Africa is expected to increase twofold (3, 4). In 2050, 80% of older people will live in low- and middle-income countries (LMIC) (1).

Recently, surgery in older patients has become a significant but overlooked public health issue in LMIC, with an increase in the older population superimposed by unmet demand for surgical care (5–7). According to a report from a low-income country, two out of five deaths among the older population might be benefited by providing access to surgical care (8). Since numerous studies from both LMIC and HIC demonstrated increased surgical demand among older people, this problem is an upcoming global issue (8–11). Also, an alarming rise in chronic diseases such as cardiac, renal, and endocrine comorbidities in the aging population makes geriatric surgery a vital component of health care (12, 13). Inevitable physiologic and anatomic aging-related changes to the body, in return affecting perioperative outcomes, made surgery for the elderly a crucial challenge in medicine (14, 15).

Previous studies established that outcomes after surgery are significantly poor in old age compared with young patients, described by increased mortality, morbidity, disability, and healthcare costs (16–18). While few studies tried to document the perioperative outcome of geriatrics in Africa, the perioperative mortality rate of older patients was reported to be 15.2% to 17.2% (19, 20). Multiple investigations reported higher mortality among geriatrics than younger, mainly attributed to comorbidity, declined physiologic reserve, and frailty (21). As the number of surgical procedures performed on elderly patients continues to rise, effective preoperative risk assessment methods are becoming increasingly important (22).

Despite postoperative mortality in geriatrics surgery being a significant healthcare burden in low-resource countries, including Ethiopia, studies are limited to planning effective methods to address the disease burden. On top of this, previously conducted studies are retrospective and used descriptive approaches, which render in identifying potential risk factors for postoperative mortality among geriatrics. As a result, the purpose of this study is to use prospective data and a survival analysis model to examine the incidence and identify risk factors for postoperative mortality among geriatrics in a low-resource country, Ethiopia.

## Methods and materials

### Study design and setting

This prospective cohort study was conducted from June 01, 2019, to June 30, 2021, at Tibebe Ghion Specialized Hospital (TGSH), Bahir Dar, Ethiopia. TGSH, one of the tertiary-level hospitals located in Bahir Dar City, North West, Ethiopia, and affiliated with Bahir Dar University, provides clinical and academic services. Bahir Dar is the capital city of Amhara regional state and is located 508 kilometers from Addis Ababa, the capital city of Ethiopia, with a population size of 318,429 in 2019.

TGSH has a wide range of patient catchment areas serving all Amhara regional state zones, the nearby zones of Oromia, and the Benshingul-Gumuz regional state. The hospital has more than 500 beds and 11 major operation rooms. Senior specialists and subspecialties doctors for all geriatric patients needing surgical interventions provide general surgery, neurosurgery, head and neck, cardiothoracic, orthopedic, maxillo-facial surgery, ear, nose, throat (ENT), and gynecological surgery at TGSH.

### Study population

During the study period, all patients aged ≥60 who had surgery in an operating room under the supervision of an anesthesia provider were eligible for inclusion. The exclusion criteria for the study was the unavailability of the patient’s cell phone for follow-up.

### Variables of the study

The study’s primary outcome was time to death in days within 28 days after surgery. For this reason, patients who died within 28 days of surgery were considered events, but those still alive on the 28th day were considered censored cases. The explanatory variables were age, gender, district, ASA physical status (23), comorbidity (coexistence of disorders in addition to a primary disease of interest), trauma, urgency of surgery, hemoglobin level, procedure type, length of surgery, anesthesia type, blood loss, and blood transfusion.

### Data collection procedure and quality

Data were collected prospectively using Research Electronic Data Capture (REDCap), an online-based data collection tool that allows offline data entry. A data collection tool previously used to record postoperative mortality in a resource-limited setting (24).

A dedicated data manager, serving as the research staff member, was responsible for collecting follow-up data and maintaining the functionality of the computerized database. The anesthesia student or provider primarily collected the initial case information using electronic tablets offline. Subsequently, the data manager completed the follow-up data, including mortality and discharge status up to 28 days, and ultimately uploaded the data to the data server using the internet. Furthermore, the data manager also ensured the completion of any missing data.

A simulation-based instruction was provided on how to utilize the electronic tool and training on the concepts of data handling. Quality improvement training was provided for the data collectors regarding ethics and challenges during clinical data collection. Data managers regularly cross-check the records with the logbook of the hospitals. A REDCap database was used to store recorded data which is secured and safe.

### Follow-up

The mortality data were collected by data managers who tracked the progress of all patients postoperatively until 28 days later. The mortality status of the patient was established using either of two methods: (1) from in-hospital data and (2) a follow-up phone call to the patient or authorized caregiver. Incorrect follow-up information, such as incorrect phone numbers, was omitted from follow-up and analysis.

### Data analysis

STATA version 17 statistical software was used for data analysis. Descriptive statistics results were presented with tables and graphs. Survival analysis models were used to estimate the incidence and identify postoperative mortality among geriatrics.

In the model, building up a non-parametric model, the Life table and Kaplan–Meier failure estimates were assessed for death probability. Log-rank test was used to examine the difference in survival between the two categorical variables. Variables found to be significant in the log-rank test were considered for Cox regression analysis. A semi-parametric Cox proportional hazard model was used to identify predictors of postoperative mortality. Before fitting the Cox regression, the assumption of proportional hazard was assessed using Schoenfeld residual and log–log plot, and the assumption was satisfied (*p*-value >0.05). Both bivariable and multivariable Cox regression was employed. Variables with *p*-value <0.2 in the bivariable Cox regression were considered for multivariable Cox regression. A *p*-value <0.05 was used to declare statistical significance.

### Ethics approval and consent to participate

This study was approved by the institutional review board (IRB) of the College of Medicine and Health Science, Bahirdar University (Reference number: 0163/2018), The need for written informed consent was waived for all study subjects by the institutional review board (IRB) of College of Medicine and Health Science, Bahirdar University and Tibebe Ghion Specialized Hospital. All methods were carried out in accordance with relevant guidelines and regulations.

## Results

### Demographic characteristics of study subjects

We captured six hundred eighteen surgical patients aged >60 during the study period. We omitted 17 records (2.83%) due to incomplete data and included 601 records in the final analysis. Most (91.34%) study subjects were middle-old, aged between 60 to 80 years, and nearly three-fourths (74.38%) were males. Regarding the zonal district of geriatrics surgical patients at TGSH, 305(50.75%), 127 (21.13%), and 91(15.14%) patients came from West Gojjam, East Gojjam, and South Gondar zones (Table 1).

**Table 1:** Demographic characteristics of the geriatric surgical patients, June 2019–June 2021.

Variable	Category	Frequency	Percentage
Age (years)	60–80	549	91.34%
	≥80	52	8.66%
Gender	Male	447	74.38%
	Female	154	25.62%
Residence (zonal district)	West Gojjam	305	50.75%
	East Gojjam	127	21.13%
	Awi	30	4.99%
	North Gondar	30	4.99%
	South Gondar	91	15.14%
	Wollo	9	1.50%
	Other#	9	1.50%

# = district including Wag-Hemera, Metekel, Oromia special zones.

### Clinical characteristics of study subjects

**Figure 1:**
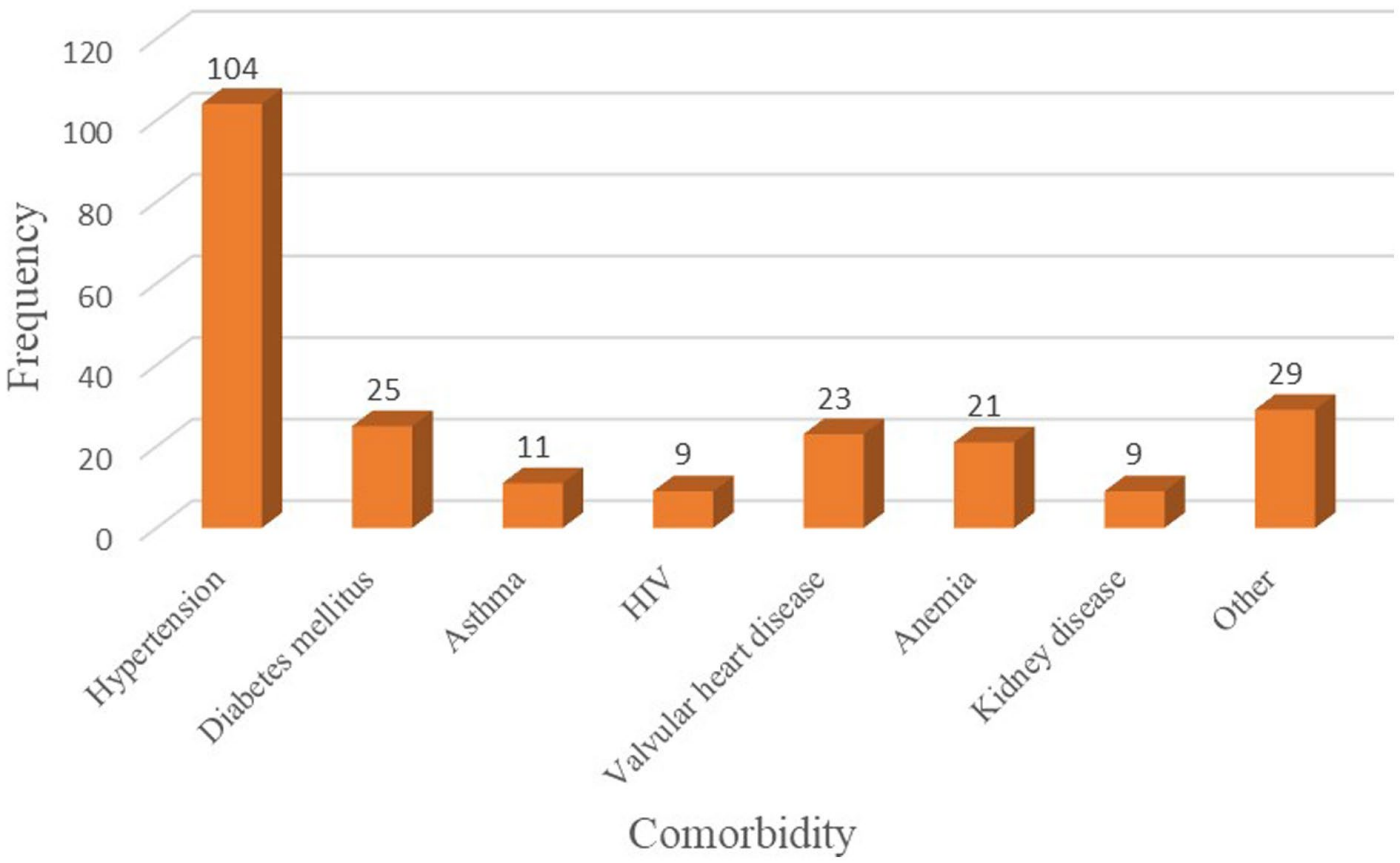
Common comorbidities among geriatric surgical patients.

Of all study subjects, 85 (14.14%) patients had ASA physical status III/IV, and 231 (38.44%) patients had some form of comorbidity. Hypertension, Diabetes Mellitus, and valvular heart disease were the most frequent comorbid conditions among old-age surgical patients (Figure 1). Nearly a quarter (22.80%) of geriatric surgical patients had a traumatic injury, and 48.92% underwent emergency surgery. Most geriatrics underwent general surgery, followed by orthopedic surgery and neurosurgery. Three hundred thirty-two (55.24%) elderly patients who have undergone surgery received general anesthesia. The median length of surgery and hemoglobin level was 85 min (60, 125) and 12.8 g/dL (11, 14), respectively. More than two-thirds (78.87%) of study subjects had an intraoperative blood loss of <500 mL, while 67 (11.15%) patients had blood transfusions intraoperatively (Table 2).

**Table 2:** Clinical characteristics of geriatric surgical patients, June 2019–June 2021.

Variable	Category	Frequency	Percentage
ASA physical status	I/II	516	85.86%
	III/IV	85	14.14%
Comorbidity	Yes	231	38.44%
	No	370	61.56%
Urgency	Elective	307	51.08%
	Emergency	294	48.92%
Trauma	Yes	137	22.80%
	No	464	77. 20%
Procedure	General surgery	170	28.29%
	Orthopedics	133	22.13%
	Neurosurgery	117	19.47%
	Other#	181	30.12%
Type of anesthesia	General	332	55.24%
	Regional	269	44.76%
Length of surgery (minute)^&^	85 (60, 125)		
Hemoglobin (g/dl)^&^	12.8 (11, 14)		
Blood loss (ml)	<500	474	78.87%
	≥500	127	21.13%
Blood transfusion	Yes	67	11.15%
	No	534	88.85%

& = median with interquartile range, # = surgeriers including head and neck, cardiothoracic, maxillo-facial, ear, nose, throat (ENT), and gynecological.

### Postoperastive mortality of geriatric patients

A total of 601 study subjects were followed for 16,189 person-day observations; 31 (5.16%) patients died, 570 (94.84%) patients were censored as 24 (3.99%) patients were lost to follow-up, and 546 (90.85%) patients survived at 28 days following surgery. The overall incidence rate of postoperative mortality among geriatric patients at TGSH was 1.91 (95% CI: 1.34, 2.72) deaths per 1,000 person-day observation. Among geriatrics with comorbidity, a high proportion of postoperative death was observed in old-age patients with valvular heart disease, anemia, and hypertension (Table 3) Concerning the time to death, the Kaplan–Meier failure curve showed that geriatric postoperative mortality increases over time (Figure 2).

**Table 3:** Impact of preoperative comorbidities on postoperative outcome, June 2019–June 2021.

Comorbidity	Total	Died	Proportion of death
Hypertension	104	14	0.13
Diabetes melilotus	25	3	0.12
Asthma	11	0	0
HIV^#^	9	0	0
Valvular heart disease	23	5	0.21
Anemia	21	3	0.14
Kidney disease	9	1	0.11
Others^&^	21	5	0.23

# = Human immunodeficiency virus; & = comorbidity including epilepsy, liver disease, psychological disease, chronic obstructive pulmonary disease.

**Figure 2:**
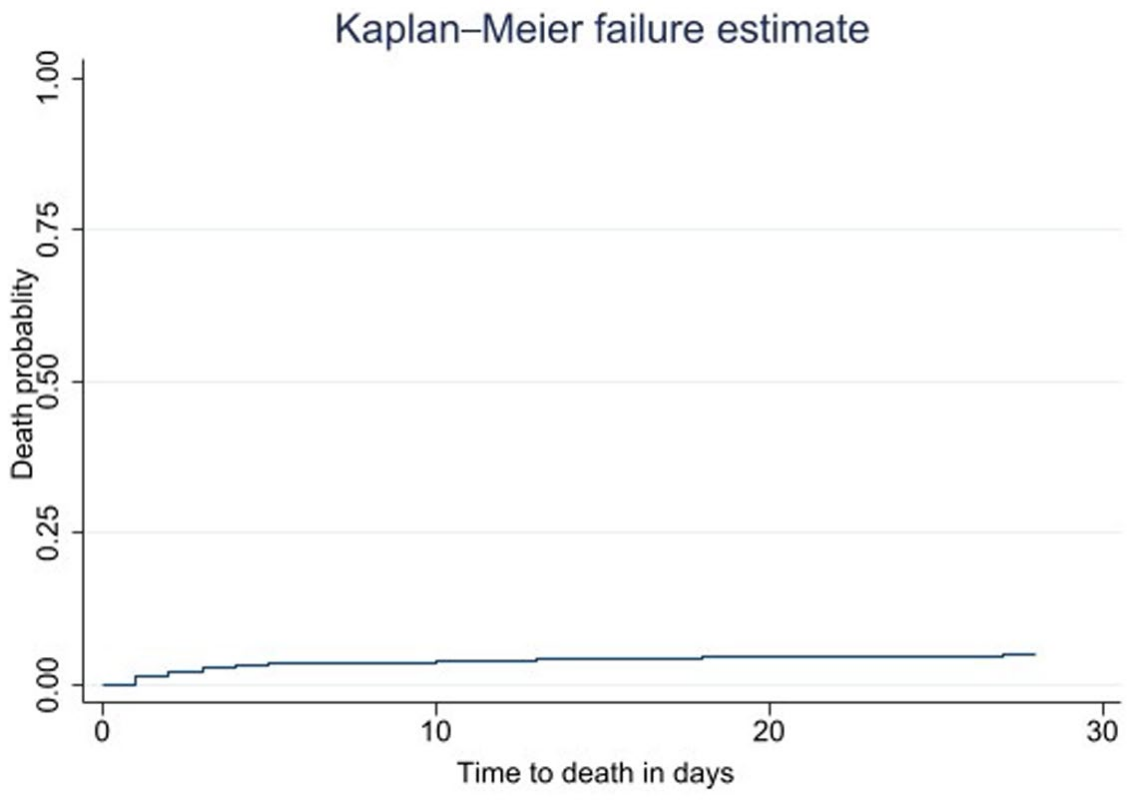
The Kaplan–Meier failure curve of postoperative geriatric mortality, Ethiopia.

### Predictors of postoperative mortality among geriatrics

The significantly identified predictors for time to death among geriatrics following surgery were age ≥ 80 years (AHR = 2.59, 95% CI: 1.05, 6.36), ASA physical status III/IV (AHR = 2.40, 95%CI 1.06, 5.43), comorbidity (AHR = 2.53, 95% CI: 1.19, 7.01), and emergency surgery (AHR = 2.92, 95% CI: 1.17, 7.27) (Table 4).

**Table 4:** Predictors of postoperative mortality among geriatrics, June 2019–June 2021.

Variable	Category	Total	Died	CHR (95% CI)	AHR (95% CI)
Age (years)	60–80	549	24	1	1
	≥80	52	7	3.14 (1.35, 7.29)	2.59 (1.05, 6.36)*
ASA physical status	I/II	516	16	1	1
	III/IV	85	15	6.09 (3.01, 12.32)	2.40 (1.06, 5.43)*
Comorbidity	Yes	231	23	4.78 (2.14, 10.69)	2.53 (1.19, 7.01)*
	No	370	8	1	1
Hypertension	Yes	104	14	2.31 (1.08, 4.91)	1.26 (0.50, 3.18)
	No	497	17	1	1
Diabetes mellitus	Yes	25	3	3.52 (1.07, 11.60)	1.55 (0.41, 5.88)
	No	576	28	1	1
Urgency	Elective	307	7	1	1
	Emergency	294	24	3.64 (1.57, 8.45)	2.92 (1.17, 7.27)*
Type of anesthesia	General	332	23	2.38 (1.07, 5.33)	1.73 (0.72, 4.36)
	Regional	269	8	1	1
Length of surgery (minute)		85 (60, 125)	88 (60, 203)	1.04 (1.03, 1.07)	1.01 (0.96, 1.11)
Hemoglobin (g/dl)		12.8 (11, 14)	11.9 (10, 14.2)	0.86 (0.74, 0.99)	0.91 (0.78, 1.06)
Transfusion	Yes	67	9	3.53 (1.62, 7.63)	1.86 (0.65, 5.31)
	No	534	22	1	1

*= p-value < 0.05; CHR, crude hazard ratio; AHR, adjusted hazard ratio.

## Discussion

Our findings, the first from a prospective study of geriatric postoperative mortality in an Ethiopian tertiary hospital, show that mortality is high and factors including age ≥ 80 years, ASA physical status, comorbidity, and emergency surgery significantly predict postoperative death among elderlies. These results are helpful as baseline information about geriatric perioperative outcomes in a resource-limited country, Ethiopia. In addition, our results enable clinicians and other stakeholders to implement evidence-based practice and prognosis-tailored services in settings with limited resources.

The overall incidence of postoperative geriatric mortality was 5.16%, with a rate of 1.91 deaths per 1,000 person-day observation, comparable to a study done in Indonesia (25). On the contrary, our result was lower than reports from Sub-Saharan Africa, with 15.2% in Tanzania (20) and 17.2% in Togo (19). The reason might be that senior surgeons and master anesthetists handle geriatric patients in the perioperative period in our setting. However, our results were higher than studies conducted in HIC (21, 26–28). The explanation for this discrepancy could be that in high-income countries, perioperative geriatric care is delivered in a well-equipped particular geriatric unit; moreover, comanagement by geriatricians and surgeons contributes to significantly lower perioperative mortality (29, 30).

Oldest-old (age ≥ 80 years) patients were observed to have a higher risk of postoperative geriatric mortality compared to middle-old (age 60–80 years) patients, corroborated by prior studies in both HIC (5, 28, 31) and LMIC (32). The possible explanation might be that the likelihood of geriatric syndromes such as delirium, frailty, functional impairment, and malnutrition increases with age, negatively affecting postoperative outcomes (33, 34). In addition, comorbid conditions are common in the oldest-old patients and increase the risk of postoperative mortality (35).

The postoperative mortality hazard was higher in patients with a higher ASA score (ASA III/IV) compared with a lower ASA score (ASA I/II). This finding aligns with studies conducted in Spain (28), Turkey (32), Japan (36), and Tanzania (20). The ability of the ASA physical status score to predict postoperative mortality is in doubt and controversial because the scoring system is highly susceptible to subjectivity (28). Higher ASA scores are related to poor physical state due to a lack of physiologic reserve to cope with stress like surgery, which may explain higher postoperative mortality (37).

This study revealed that geriatrics with comorbidity had an increased risk of postoperative death consistent with previous reports (38, 39). The reason might be that preoperative comorbidities increase the risk of life-threatening postoperative complications such as myocardial infarction, intracerebral hemorrhage, and thromboembolic events, which increase mortality risk (38). In addition, our study revealed the high prevalence of comorbid medical illness among geriatrics, necessitating targeted plans and actions to minimize comorbidities and their related consequences. Comprehensive geriatric assessment aims to assess and enhance older patients’ physical, psychological, functional, and social aspects. The implementation of this approach helps improve long-term outcomes for this population (15). This study also highlighted that the hazard of postoperative mortality was high among geriatrics who underwent emergency surgery, in agreement with other studies (25, 31, 36). The explanation might be that symptoms such as hemorrhage, acidosis, and tissue hypoperfusion may suggest an advanced stage of diseases in patients who presented for emergency surgery, contributing to poor survival (36).

As a strength, we used prospective data, adequate sample size, and a survival analysis model to identify predictors. Furthermore, we followed patients until 28 postoperative days, which is acceptable for capturing mortality data. To mention some limitation, this study was a single center. This study also did not assess some relevant variables that might affect older patients’ postoperative mortality, such as delirium, polypharmacy and nutritional variables. Moreover, we assume deaths recorded during the 28-day follow-up related to surgery which affects the incidence.

## Conclusion

This cohort study showed a high incidence of postoperative mortality among geriatrics at TGSH. Age ≥ 80 years, ASA physical status III/IV, comorbidity, and emergency surgery were significant predictors of postoperative mortality among geriatrics. Based on our results, health professionals involved in perioperative care for geriatric patients should give target-specific interventions for the identified group of patients.

## Data availability statement

The raw data supporting the conclusions of this article will be made available by the authors, without undue reservation.

## Ethics statement

This study was approved by the institutional review board (IRB) of the College of Medicine and Health Science, Bahirdar University, Ethiopia. The studies were conducted in accordance with the local legislation and institutional requirements. The ethics committee/institutional review board waived the requirement of written informed consent for participation from the participants or the participants’ legal guardians/next of kin because This study hold a very minimal risk to study subjects and might include subjects who are very critically ill and unconscious during the time of enrolment to the study.

## Author contributions

AE: took part in conceptualization, methodology, formal analysis, investigation, resources, data curation, writing – original manuscript draft, writing – review & editing, and visualization. FK: took part in methodology, formal analysis, investigation, writing review & editing, and visualization. MM: manuscript writing, paper revision, editing, and methodology. All authors contributed to the article and approved the submitted version.

## Abbreviations

AHR: adjusted hazard ratio
ASA: American society of anesthesiologist
CHR: crude hazard ratio
HIC: high-income countries
IQR: interquartile range
LMIC: low-and-middle-income countries
TGSH: tibebe ghion specialized hospital
