# Supplementary material for: Perioperative mortality among geriatric patients in Ethiopia: a prospective cohort study
Source: Front Med (Lausanne). 2023 Nov 2;10:1220024. doi: 10.3389/fmed.2023.1220024 (PMC10651902; doi:10.3389/fmed.2023.1220024)
Supplement: Supplementary file 1 [file Table_1.docx]

Supplementary table 1: Log-rank test for the predictors of geriatrics postoperative mortality

| Variable |  | p-value |
| --- | --- | --- |
| Age |  | <0.0001 |
| Gender |  | 0.6821 |
| Residence |  | 0.2995 |
| ASA physical status |  | <0.0001 |
| Comorbidity |  | <0.0001 |
| Hypertension |  | 0.0002 |
| Diabetes Melliteus |  | 0.011 |
| Valvular heart disease |  | 0.0811 |
| Urgency |  | 0.0012 |
| Trauma |  | 0.4030 |
| Procedure |  | 0.1948 |
| Type of anesthesia |  | 0.0287 |
| Blood loss |  | 0.8243 |
| Blood transfusion |  | 0.0007 |

Supplementary table 2: Schoenfeld residua test for checking proportional hazard assumption for the incidence of postoperative mortality and its predictors among geriatrics in Ethiopia

| Variable | rho | df | p-value |
| --- | --- | --- | --- |
| Age | 0.44701 | 1 | 0.0901 |
| ASA physical status | 0.05448 | 1 | 0.6927 |
| Comorbidity | 0.06335 | 1 | 0.6946 |
| Hypertension | 0.0548 | 1 | 0.118 |
| Diabetes Mellitus | 0.0874 | 1 | 0.128 |
| Urgency | 0.34856 | 1 | 0.0824 |
| Type of anesthesia | 0.12662 | 1 | 0.4631 |
| Length of surgery | 0.04765 | 1 | 0.7876 |
| Hemoglobin | -0.01642 | 1 | 0.9188 |
| Transfusion | 0.30517 | 1 | 0.1055 |
|  | Global test | 8 | 0.2482 |
